# The MAMBO Framework and the Future of Modern Perfusion: Artificial Intelligence, Cognitive Ergonomics, and Sustainable Workflow Integration

**DOI:** 10.7759/cureus.110501

**Published:** 2026-06-08

**Authors:** Ignazio Condello, Youssef El Dsouki

**Affiliations:** 1 Cardiac Surgery, University of Insubria, Varese, ITA; 2 Cardiac Surgery, Maastricht University Medical Centre, Maastricht, NLD

**Keywords:** artificial intelligence, blood oxygenation, cardiopulmonary bypass, framework, perfusion

## Abstract

Artificial intelligence (AI), predictive analytics, and digital health technologies are progressively reshaping extracorporeal circulation and perioperative cardiovascular care. In this evolving landscape, cardiopulmonary bypass management is transitioning from a predominantly technical discipline to a model of precision extracorporeal medicine that integrates physiologic optimization, cognitive ergonomics, and workflow sustainability. The Modern Advanced Management of Blood Oxygenation (MAMBO) Framework was originally developed as a physiologic strategy centered on monitoring, adaptation, management, balance, and optimization during cardiopulmonary bypass. However, its applications may extend beyond patient physiology alone. Increasing evidence highlights the growing impact of cognitive workload, operator stress, alarm fatigue, and organizational complexity in contemporary perfusion practice, while advances in AI-assisted monitoring, explainable machine learning, and predictive analytics are creating new opportunities to enhance both clinical performance and professional sustainability. In this editorial perspective, we discuss the expanded role of the MAMBO Framework as a human-centered and workflow-oriented model for the future of perfusion science. We explore how AI-supported systems and adaptive decision-support strategies may contribute not only to physiologic precision and the optimization of oxygen delivery but also to reduced cognitive overload, improved situational awareness, and greater efficiency in multidisciplinary workflows. The convergence of AI, cognitive ergonomics, and precision perfusion may ultimately redefine the role of the modern perfusionist, promoting a more adaptive, sustainable, and data-driven approach to cardiovascular care.

## Editorial

Introduction

Cardiopulmonary bypass (CPB) management is undergoing a profound transformation driven by the progressive integration of artificial intelligence (AI), predictive analytics, digital health technologies, and precision physiologic monitoring into perioperative cardiovascular medicine [1]. Traditionally, perfusion practice has been considered a highly technical discipline centered on maintenance of extracorporeal circulation, oxygen delivery, and hemodynamic support during cardiac surgery. However, the increasing complexity of modern cardiovascular procedures has progressively expanded the role of the perfusionist from technical operator to physiologic strategist. Recent machine learning models have demonstrated the ability to predict perioperative complications by integrating demographic, metabolic, institutional, and intraoperative variables into dynamic risk prediction systems [2]. Importantly, many determinants identified by explainable AI algorithms, including oxygen delivery thresholds, perfusion adequacy, renal protection, and hemodynamic optimization, remain directly influenced by extracorporeal circulation management [3]. This observation suggests that future improvements in cardiac surgical outcomes may depend not only on more accurate predictive models but also on the development of operational frameworks capable of translating predictive information into real-time physiologic interventions. In parallel, increasing attention has been directed toward the cognitive and organizational dimensions of perfusion practice [4]. Modern operating rooms are characterized by high-density information streams, technological complexity, alarm fatigue, and sustained cognitive demand [5]. Recent studies investigating perfusionists’ workload and stress through digital biomarkers and physiologic monitoring have demonstrated that cognitive burden may significantly influence intraoperative performance and decision-making stability [3]. Within this evolving landscape, the Modern Advanced Management of Blood Oxygenation (MAMBO) Framework may represent a broader conceptual model integrating physiologic precision, workflow optimization, cognitive ergonomics, and AI-assisted decision support. Rather than functioning solely as a technical protocol, the MAMBO Framework may be interpreted as a flexible and adaptive philosophy for modern perfusion practice. The purpose of this editorial is to discuss the potential role of the MAMBO Framework in the future of extracorporeal circulation, with particular attention on AI integration, cognitive stress reduction, workflow sustainability, and the evolving role of the modern perfusionist.

The digital transformation of perfusion science

Cardiovascular surgery is progressively transitioning toward an integrated digital ecosystem in which AI, predictive analytics, physiologic monitoring, and data-driven decision support are reshaping perioperative management. Within this transformation, extracorporeal circulation is no longer viewed simply as a technical component of surgery but increasingly as a dynamic physiologic intervention capable of influencing short- and long-term patient outcomes. Modern perfusion, therefore, requires not only technical expertise but also continuous interpretation of complex physiologic interactions occurring in real time. Recent developments in machine learning have demonstrated the capacity of predictive systems to identify intraoperative risk profiles by integrating demographic, hemodynamic, metabolic, institutional, and perfusion-related variables [1-3]. However, prediction alone cannot improve outcomes unless predictive information is translated into actionable physiologic strategies. This concept represents one of the central philosophical foundations of the MAMBO Framework. The evolution of CPB management is entering a new phase in which AI, predictive analytics, digital biomarkers, and precision perfusion are progressively converging into a unified physiologic ecosystem. Recent evidence has demonstrated that extracorporeal circulation is no longer simply a technical support platform; rather, it is a dynamic clinical environment in which oxygen delivery, hemodynamic stability, cognitive workload, and team interaction continuously influence patient outcomes [1-4]. In this context, the MAMBO Framework should not only be interpreted exclusively as a technological model for perfusion optimization but also as a transversal organizational and human-centered strategy capable of improving workflow, reducing operator stress, and supporting inclusivity within the perfusion workforce. Over the last decade, machine learning and AI-assisted systems have transformed perioperative risk stratification by integrating physiologic variables, institutional characteristics, and intraoperative parameters into adaptive predictive models [1-3]. Importantly, many determinants identified by explainable AI systems are directly linked to perfusion practice, including oxygen delivery thresholds, perfusion pressure, renal protection, metabolic balance, and tissue perfusion adequacy [5]. This convergence reinforces the concept that modern perfusion is becoming a form of precision extracorporeal medicine. However, predictive capability alone cannot improve outcomes unless prediction is translated into actionable physiologic management. The real challenge is therefore operational: how can modern perfusion teams transform continuous data streams into safer, less stressful, and more efficient intraoperative decision-making? In our opinion, this is precisely where the MAMBO Framework acquires its broader significance.

MAMBO as a human-centered perfusion philosophy

The five interconnected domains of MAMBO, Monitoring, Adaptation, Management, Balance, and Optimization, can be interpreted not only as physiologic pillars of extracorporeal circulation but also as organizational principles capable of enhancing cognitive ergonomics during CPB. Rather than functioning as a rigid protocol, the MAMBO Framework should be interpreted as a flexible operational philosophy designed to integrate physiologic precision with workflow efficiency and human-centered clinical practice. In modern operating rooms characterized by increasing technological density and continuous data acquisition, perfusionists are frequently required to process large volumes of information under significant time pressure. Consequently, systems capable of simplifying interpretation and prioritizing actionable information may become increasingly important for maintaining procedural safety and decision-making stability. The integration of AI-assisted systems into perfusion practice may therefore serve a dual purpose: improving patient-specific physiologic optimization while simultaneously supporting the cognitive performance of clinicians [4].

Monitoring refers not only to patient physiologic surveillance but also to the continuous assessment of workload, cognitive stress, and team situational awareness. Emerging evidence suggests that perfusionists experience substantial acute cognitive load during cardiac surgery, particularly during critical phases of CPB and unexpected failure-mode scenarios [5]. Digital biomarkers, physiologic stress monitoring, and AI-assisted dashboards may therefore become essential tools for recognizing periods of excessive cognitive burden before human performance deteriorates [3].

Adaptation represents the ability of both clinicians and systems to dynamically respond to physiologic instability and workflow variability. AI-guided systems may reduce information overload by prioritizing clinically relevant variables and generating predictive alerts before critical deterioration occurs [2,3]. Such adaptive support mechanisms may reduce mental fatigue, improve confidence in decision-making, and facilitate more stable team communication.

Management extends beyond perfusion flow regulation and oxygen transport optimization. It also encompasses the orchestration of workflow, communication, alarm management, and multidisciplinary coordination inside the operating room. Modern perfusion environments are increasingly characterized by high-density data streams and technological complexity. Structured frameworks such as the MAMBO Framework may contribute to reducing unnecessary variability and creating more standardized cognitive pathways during stressful situations.

Balance should be interpreted as both physiologic and human balance. While maintaining equilibrium between oxygen delivery and metabolic demand remains central to CPB management [5], maintaining psychological balance among perfusion professionals is becoming equally important. Chronic stress, prolonged vigilance, cognitive overload, and fatigue represent underrecognized threats to both operator well-being and patient safety. Human-centered perfusion models integrating AI support, ergonomic workflows, and predictive analytics may therefore improve not only clinical outcomes but also occupational sustainability [4].

Finally, Optimization should not be limited to patient-specific physiologic targets. Optimization must also include workflow simplification, reduction of avoidable stressors, enhancement of communication dynamics, and improved organizational sustainability within perfusion teams.

Cognitive stress and workflow sustainability

One of the emerging themes in modern perfusion science is the recognition that cognitive workload and operator stress directly influence clinical performance [3]. During CPB, perfusionists are exposed to prolonged periods of sustained vigilance, rapid decision-making, alarm management, and complex multidisciplinary interaction. These demands may become even more pronounced during emergency situations, unexpected physiologic instability, or equipment-related failure scenarios. Recent studies investigating digital biomarkers and cognitive load monitoring have highlighted the possibility of objectively quantifying stress and mental workload during cardiac surgery [4]. This represents a particularly important evolution because traditional assessments of operator fatigue and cognitive burden have historically relied on subjective reporting rather than physiologic or data-driven measurements. Within this context, the MAMBO Framework may contribute to the development of more sustainable perfusion environments by promoting adaptive monitoring systems, predictive alerts, workflow standardization, and reduction of unnecessary informational overload. Such strategies may facilitate more stable situational awareness and reduce cognitive fragmentation during critical intraoperative phases. Importantly, improving workflow sustainability is not exclusively related to technological innovation. It also involves redesigning the interaction between clinicians, machines, physiologic data, and decision-support systems. Human-centered perfusion models capable of integrating automation without reducing clinical autonomy may therefore represent one of the most relevant future directions for extracorporeal circulation. The perfusion workforce is evolving globally, with increasing attention directed toward professional well-being, inclusivity, workforce sustainability, and long-term retention [5]. Modern perfusion environments are characterized by prolonged vigilance, high cognitive demand, technological complexity, and continuous decision-making pressure. These factors may contribute to occupational stress, fatigue, burnout, and reduced workforce sustainability across the profession. Consequently, there is growing recognition that future perfusion models should support not only physiologic precision but also healthier and more sustainable working environments for all professionals involved. Within this perspective, the MAMBO Framework may offer an important transversal contribution. By integrating AI-assisted decision support, predictive monitoring, cognitive workload assessment, and workflow standardization, the MAMBO Framework has the potential to create more sustainable and supportive professional environments. Reduced alarm fatigue, more intuitive data integration, and adaptive perfusion platforms may improve operational efficiency while simultaneously lowering psychological pressure during complex procedures. Importantly, these advantages extend across the entire multidisciplinary perfusion community. A more ergonomic and cognitively supported operating room environment may improve communication, resilience, decision-making stability, and professional sustainability while reducing unnecessary stressors during complex procedures. In this context, human-centered perfusion systems may contribute to a more inclusive, efficient, and collaborative workflow culture.

Redefining the role of the modern perfusionist

The future perfusionist may therefore evolve from a primarily technical operator into a highly integrated physiologic strategist supported by digital ecosystems, AI-assisted analytics, and human-centered workflow architectures [4]. This transformation will likely require new competencies extending beyond conventional extracorporeal circulation management. Future perfusion practice may increasingly involve interpretation of predictive algorithms, management of AI-assisted systems, understanding of digital biomarkers, and integration of physiologic analytics into real-time clinical decision-making. At the same time, the modernization of perfusion should preserve the central role of human expertise. AI should not replace clinical judgment but rather augment the clinician’s ability to recognize physiologic deterioration, prioritize interventions, and reduce avoidable cognitive overload. In this perspective, the relationship between technology and perfusion practice should be viewed as synergistic rather than competitive. The convergence of physiologic precision, predictive analytics, cognitive ergonomics, and workflow optimization may therefore define the next generation of extracorporeal circulation management. In this evolving scenario, precision perfusion should progress in parallel with precision workforce management. The next frontier of extracorporeal circulation may not depend solely on improving predictive algorithms but rather on building integrated systems capable of simultaneously optimizing patient physiology, reducing cognitive stress, supporting professional well-being, and enhancing organizational sustainability and workflow efficiency. In our opinion, the true innovation of the MAMBO Framework is not limited to technology itself. Its greatest potential may reside in its ability to harmonize physiologic precision, AI, cognitive ergonomics, and human sustainability into a unified model for the modern perfusion era.

Conclusions

The evolution of extracorporeal circulation is progressively redefining the boundaries of modern perfusion science. AI, predictive analytics, digital biomarkers, and adaptive monitoring systems are transforming CPB management from a predominantly technical activity into an integrated model of precision physiologic intervention. Within this context, the MAMBO Framework may represent more than a strategy for oxygen delivery optimization. Its broader significance may reside in its ability to integrate physiologic precision, workflow sustainability, cognitive ergonomics, and human-centered decision support into a unified operational philosophy. Future perfusion systems will likely require not only more accurate predictive algorithms but also organizational models capable of reducing cognitive overload, improving situational awareness, facilitating multidisciplinary communication, and supporting more sustainable intraoperative workflows. The integration of AI-assisted systems should therefore be viewed not as a replacement for clinical expertise but as a mechanism for augmenting human performance and reinforcing physiologic decision-making. The modern perfusionist may increasingly evolve into a data-driven physiologic strategist operating within digitally integrated clinical ecosystems. In this scenario, the convergence of AI and extracorporeal circulation management may contribute to safer procedures, improved workflow efficiency, and more resilient perioperative care systems. The MAMBO Framework may therefore represent an important conceptual step toward the future of precision perfusion and the next generation of cardiovascular surgical care.
